# Skin Staples: A Safe Technique for Securing Mesh in Lichtensteins Hernioplasty as Compared to Suture

**DOI:** 10.1155/2014/958634

**Published:** 2014-04-03

**Authors:** Anand Munghate, Sushil Mittal, Harnam Singh, Gurpreet Singh, Manish Yadav

**Affiliations:** Department of Surgery, Government Medical College, Patiala 147001, India

## Abstract

*Background.* Lichtenstein tension free repair is the most commonly used technique due to cost effectiveness, low recurrence rate, and better patient satisfaction. This study was done to compare the duration of surgery and postoperative outcome of securing mesh with skin staples versus polypropylene sutures in Lichtenstein hernia repair. * Materials and Methods.* A total of 96 patients with inguinal hernia undergoing Lichtenstein mesh repair were randomly assigned into two groups. The mesh was secured either by using skin staples (group I) or polypropylene sutures (group II). *Results.* The operation time was significantly reduced from mesh insertion to completion of skin closure in group I (mean 20.7 min) as compared to group II (mean 32.7 min) with significant *P* value (*P* < 0.0001) and less complication rate in group I as compared to group II. *Conclusion.* Mesh fixation with skin staples is as effective as conventional sutures with added advantage of significant reduction in the operating time and complications or recurrence. The staples can be applied much more quickly than sutures for fixing the mesh, thus saving the operating time. Infection rate is significantly decreased with staples.

## 1. Introduction 

Hernia is defined as a protrusion of a viscus or a part of viscus through an abnormal opening in the wall of its containing cavity. The most frequent of all hernia is inguinal hernia, which occurs in 73% of all the hernia cases and is 20 times more frequent in males than females [1].

Lichtenstein et al. (1989) reported that excessive tension on the suture line resulted in the high recurrence rate after the primary repair. In 1989, Lichtenstein et al. concluded that with tension free mesh repair of hernia, recurrence can be completely avoided. Although many new techniques are available today for hernia repair (plug and patch, TEP, TAPP, PHS), Lichtenstein tension free repair is the most commonly used technique due to cost effectiveness, low recurrence rate, and better patient satisfaction [2]. The Lichtenstein repair takes into account the important factors identified in the successful outcome of hernia operation—supplementing the strength of transversalis fascia and a tension free repair. The only disadvantage of the mesh operation is that it requires the use of prosthetic material with attendant risk of infection. Any modification which reduces this threat would be useful.

The main cause for recurrence of hernia is “Suture line tension” brought by suturing of overcasting between annular and ligamentous flap which are not normally in apposition. Needle hole and the tension created by suture material on tissue destroy the valuable sling and shutter mechanism [3, 4].

The latest trial in this aspect is securing mesh with use of skin staples instead of the usual polypropylene sutures. Staples are applied from a proximate plus MD (multidirectional) release skin stapler. Staples are quick to use and reduce the operating time and minimize the risk of wound infection [5].

## 2. Materials and Methods

**Figure 1:**
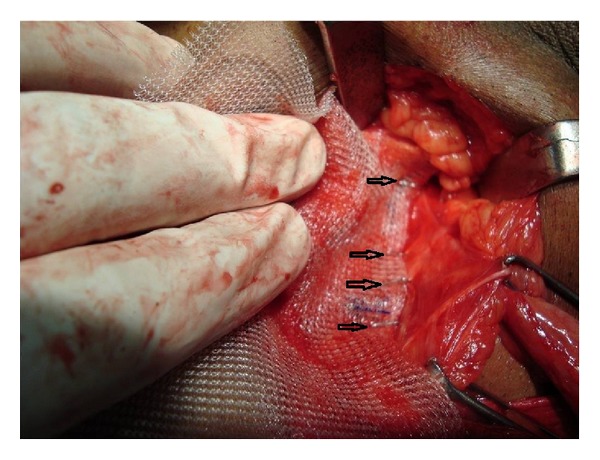
Arrow head showing staples along the inguinal ligament.

**Figure 2:**
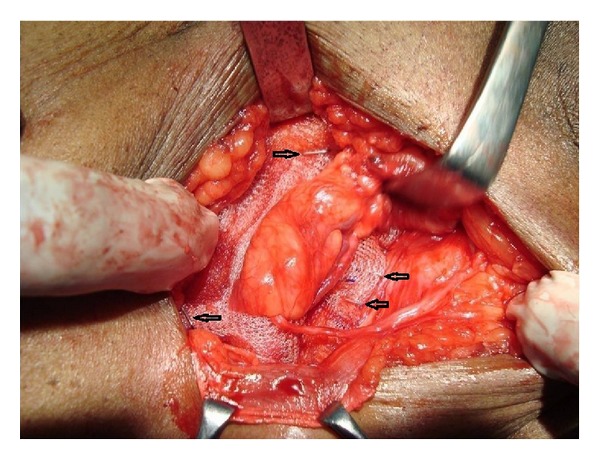
Arrow head showing staples along the inguinal ligament laterally and conjoint tendon and fascia transversalis medially.

This study was carried out in the Department of Surgery, Government Medical College and Rajindra Hospital Patiala (India), from June 2011 to June 2013. Ninety-six adult patients (>18 yrs) with primary inguinal hernias were entered into the trial, all as elective cases. Informed consent was obtained. Patients were randomized either to the group I (where the mesh was secured with staples) or the group II (where the mesh was sutured with polypropylene sutures). Two patients in each group underwent bilateral inguinal hernia repair thus making 50 mesh repairs in each group. Most of the operations were done under spinal anaesthesia. A single dose of intravenous cefotaxime 1 gram was administered 1 hour prior to surgery. Direct hernia sacs were plicated unless very small, when they were reduced unopened. Small indirect sacs were dissected from the spermatic cord and then divided and transfixed and distal part was excised. A sheet of polypropylene mesh (11 × 6 cm) was cut to shape and laid over the posterior wall of the inguinal canal so that it overlapped the pubic tubercle by at least 1 cm medially so that sheet can cover superiorly over the conjoint tendon and to a point at least 2 cm lateral to the internal ring. In group II this was fixed in position by interrupted sutures of 2/0 Prolene (Ethicon) along the inguinal ligament inferiorly from the pubic tubercle to the lateral edge of the mesh. Interrupted polypropylene sutures were then placed medially and superiorly into the internal oblique and transversalis muscles. The spermatic cord was passed through a slit in the mesh. Lateral to it, the overlapping free edges of the mesh were sutured together with two interrupted polypropylene sutures. In group I the positioning of the mesh was identical but a Proximate Plus MD (multidirectional) release Skin Stapler (Ethicon) containing 35 preloaded stainless steel staples was used to secure it. A staple was placed into the pubic tubercle with between three and four staples along the inguinal ligament placed 1-2 cm apart (Figure 1). Further two to three staples were placed in the internal oblique and transversalis muscle medially and superiorly (Figure 2) and the overlapping free edges of the mesh were stapled together with two staples lateral to the cord. In both groups the external oblique aponeurosis was closed with a continuous suture of 2/0 Prolene (Ethicon) and the subcutaneous tissue were then approximated with 2/0 vicryl. Skin closure was completed in group II using interrupted sutures of 3/0 Ethilon (Ethicon), which were removed 7 days after surgery. In group I skin closure was completed using staples from the same staple gun and these were removed 7 days after operation. The time taken from the skin incision to the beginning of the mesh insertion and from the beginning of the mesh insertion to completion of skin closure was recorded to the nearest 30 seconds. Antibiotics were given postoperatively to all patients for one week. Postoperative patients were made ambulatory day after surgery. Normal activity was permitted a week after. Strenuous exercise was discouraged for a month. Postoperative complications like infection, hematoma, requiring drainage or in-patient admission, pain significant to cause alteration in life style (assessed by visual analogue scale), noninfectious urinary complications including acute urinary retention that prolonged the hospital stay, postoperative ileus, and other miscellaneous complications were noted daily. Patients were discharged as soon as possible depending on the postoperative condition of the patient.

Patients were called for followup in out-patient department on 7th postoperative day, 2-3 weeks postoperatively, 1-2 months later, and then for further follow-up till 12–18 months. Check up for any complication and recurrence was carried out in detail and the observations were recorded.

Statistical analysis was performed using the Student's unpaired *t*-test.

## 3. Results 

**Figure 3:**
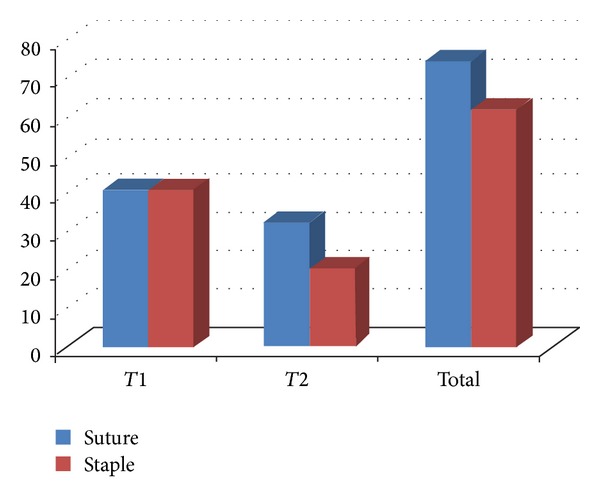
According to time variations of both groups in minutes, *T*1 is the time taken from the skin incision to the beginning of the mesh insertion. *T*2 is the time taken from the beginning of the mesh insertion to completion of skin closure.

There were 50 patients in each group and results were compared in terms of operative time and complications (Figure 3 and Tables 1 and 2).

## 4. Discussion

The treatment of inguinal hernia has evolved over the past 150 years from truss support with operation reserved for life-threatening situations to elective outpatient repair [6]. Pure tissue repairs have suture line after closure, which is under tension because the defect edges are approximated instead of being bridged. Suture line tension is at the heart of failed hernia repair and solving this problem would largely eliminate the recurrence [7]. Excessive tension on the suture line and the surrounding tissue leads to tissue ischemia and suture cut-out leading to recurrence [8].

In tension free or mesh based repair, synthetic mesh is usually used to strengthen the transversalis fascia to create a strong and tensionless repair [9]. Open mesh hernioplasty appears to be gold standard when managing inguinal hernia [10].

Lichtenstein technique of inguinal hernia repair has been proved to be an effective and safe method with low recurrence rate. Surgeons use it successfully with good results. The first author to describe staple modification to this technique, Egger et al. [11], emphasized the advantage of shorter operating time. Mills et al. [7] prospectively studied 50 patients that were operated using sutures or staples.

The main advantage with application of staples for securing the mesh in Lichtenstein repair is reduction in the operative time. Difference of 12 minutes was found between the groups I and II which was significant (*P* < 0.001). Thus, staples can be applied much more quickly than sutures hence saving the operating time, reducing tissue handling, reducing the risk of wound infection, and also reducing the risk associated with prolonged anaesthesia [7]. Similar results were also shown by Garg et al. [5].

*Complication*

*(A) Intraoperative Complications.* There was no intraoperative or immediate postoperative haemorrhage or any bladder/bowel/neurovascular injury in the present study. Gould suggested it is safe to staple the mesh a little higher up on the inguinal ligament than one might with suture [12]. Mills et al. also said that the risk of damage to major underlying vessel may be less than with insertion of conventional sutures. They suggested accurate staple placement is facilitated by the design of the stapler whose head rotates by 360° degree there by allowing for maximum visibility and improved access [7]. No other intraoperative complications were seen.

*(B) Postoperative Complications*

*(I) Urinary Retention*. There was no significant difference in the incidence of urinary retention in the two groups.

*(II) Wound Infection.* Wound infection is a major cause of hernia recurrence [2]. In the present study, 12 (24%) patients in control group II and 2 (4%) patient in study group I developed wound infection. 6 (12%) patients had serosanguinous discharge, 4 (8%) developed wound gaping and 2 (4%) had minimal discharge in the control group II. The infection rate was significantly higher in the control group II. van der Zwaal et al. interestingly reported that there was no postoperative wound infection. They suggested that it could possibly be attributed to the inert coating covering the stainless steel staples. Thus, it may be inferred that rate of wound infection is significantly less with the use of staples but further studies and patients are needed to confirm it [13].

*(III) Stitch Abscess.* 4 (8%) patients presented with stitch abscess in suture group II but there was no such complication in the staple group I.

*(IV) Wound Seroma*. In the present study, 6 (12%) patients in group II and none in group I presented with such complication. All of them resolved within 2-3 weeks with conservative management. There was no any specific indication for putting drainage. Garg et al. were the only authors to report this complication in both the groups but the seroma formation was almost equal in both groups and no significant difference was observed between the two groups. The swelling and induration of wound were transient and settled without intervention [5].

*(V) Scrotal Edema/Hematoma*. In the present study, 2 (4%) patients in group II presented with scrotal swelling and scrotal hematoma each. Swelling was managed conservatively and hematoma was aspirated. No such complication was found in staple group I.

*(VI) Nerve Entrapment*. In the present study, none of the patients in either group presented with such a complication, showing there is no increased risk of entrapment neuropathy with the use of staples [13]. Kingsnorth stated that the use of staples in the laparoscopic inguinal hernia repair can damage the nerves and small blood vessel with harmful consequences due to relatively unsighted application of staples [14, 15]. Kingsnorth also stated that the use of staples is not without the risk of entrapment neuropathy; in early case series of laparoscopic hernia repair, this complication was not reported, but with the use of stapler in later series nerve injuries began to be described [16].

*(VII) Postoperative Pain*. In the present study, no significant difference was seen in the postoperative pain in both groups. Mills et al. have reported that there was no difference in pain score between the two groups. Garg et al. also stated that there was no difference in the pain duration in both groups of their study [5, 7]. van der Zwaal et al. also reported that pain scores were similar in both the groups [13].

*(VIII) Recurrence Rate.* No early recurrence was seen in either group. A very striking finding by van der Zwaal et al. was the high recurrence rate in the suture group II-11% as compared to 1% in the staple group I. Other authors have reported recurrence rate of 0.5–3.7% in the traditional Lichtenstein repair procedure. It is known that recurrent inguinal hernia occur in the medial side. Therefore, it is important to position the mesh 1 cm medial to the pubic bone. Furthermore, a tension free position of the mesh is advocated. Their hypothesis was that staple fixation is able to create a more tension free position of the mesh as compared to sutures, because sutures are more prone to tensioning than staples [13]. Thus, there is some evidence that securing the mesh with staples instead of sutures might reduce the recurrence rate. But a detailed study with prolonged follow-up is required to comment accurately on the recurrence rate of inguinal hernia in both groups.

The median duration of hospital stay was 3 (2–6) days for both groups. Longest period of stay in suture group was 6 days and in staple group was 5 days. The use of skin staples was cost effective as compared to sutures as cost of staples was about Rs 550, which with proper sterilisation can be used for 3-4 patients in comparison to sutures, Rs 470 per patient (Propylene 2-0 Rs 350 + Silk 2-0 Cutting Rs 120).

## 5. Conclusion

The technique of Lichtenstein tension-free repair is simple, relatively easier to learn, and less technically demanding. In our study, it was concluded that the staples can be applied much more quickly than sutures for mesh fixation thus saving the operating time. This technique of mesh fixation is as effective as conventional fixation with polypropylene sutures. The stapler placement with skin stapler provides good penetration into the tissue with secure fixation of the mesh, making this method technically easier. Infection rate is significantly decreased with use of staples. The use of staples is not associated with any increase in postoperative pain and is not associated with any increase in complications as compared to the use of sutures. The use of staples is cost effective in comparison to the suture.

Thus, in our opinion, staples are a far better option for fixation of mesh as compared to conventional sutures. This study needs to be evaluated further in a larger group of patients to explore the impact of reduced operative time on postoperative complications and recurrence rate.

## Figures and Tables

**Table 1:** Comparison of operative time one (*T*1), operative time two (*T*2), and total operative time (TOT) in two groups.

S. no.	Suture			Staple		
	*T*1	*T*2	TOT	*T*1	*T*2	TOT
Mean	41.3	32.7∗	74.0∗∗	41.3	20.7∗	62.0∗∗
SD	8.8	8.3	16.2	9.0	6.9	14.4

*P < 0.0001; **P = 0.007.

**Table 2:** Comparison of various complications in two groups.

	Suture group II *n* = 50	Stapler group I *n* = 50
Operative complications		
Haemorrhage	0	0
Bowel/bladder/neurovascular injury	0	0
Any other complications	0	0
Postoperative complications (general)		
Pulmonary	0	0
Fever	0	0
Urinary retention	4 (8%)	6 (12%)
Bowel obstruction	0	0
Postoperative complications (specific)		
Wound ecchymosis	0	0
Wound infection	12 (24%)	2 (4%)
Wound hematoma	0	0
Wound seroma	6 (12%)	0
Wound abscess	0	0
Scrotal oedema	2 (4%)	0
Scrotal hematoma	0	0
Scrotal abscess	0	0
